# Sustainable Strategies for Synthesizing Lignin-Incorporated Bio-Based Waterborne Polyurethane with Tunable Characteristics

**DOI:** 10.3390/polym15193987

**Published:** 2023-10-04

**Authors:** Bo Min Kim, Jin Sil Choi, Sunjin Jang, Hyeji Park, Seung Yeol Lee, Joonhoo Jung, Jaehyeung Park

**Affiliations:** 1 Department of Carbon and Fiber Composite Materials, Kyungpook National University, Daegu 41566, Republic of Korea; kbm812@naver.com; 2 Department of Plant Medicine, Kyungpook National University, Daegu 41566, Republic of Korealeesy1123@knu.ac.kr (S.Y.L.); 3 Department of Biofibers and Biomaterial Science, Kyungpook National University, Daegu 41566, Republic of Koreayuwe6633@naver.com (H.P.); 4 ANPOLY Inc., Pohang 37666, Republic of Korea; jjung@anpolyinc.com

**Keywords:** bio-based polyurethane, waterborne polyurethane, lignin, castor oil, sustainability

## Abstract

In this study, we introduce a novel approach for synthesizing lignin-incorporated castor-oil-based cationic waterborne polyurethane (CWPU-LX), diverging significantly from conventional waterborne polyurethane dispersion synthesis methods. Our innovative method efficiently reduces the required solvent quantity for CWPU-LX synthesis to approximately 50% of that employed in traditional WBPU experimental procedures. By incorporating lignin into the polyurethane matrix using this efficient and reduced-solvent method, CWPU-LX demonstrates enhanced properties, rendering it a promising material for diverse applications. Dynamic interactions between lignin and polyurethane molecules contribute to improved mechanical properties, enhanced thermal stability, and increased solvent resistance. Dynamic interactions between lignin and polyurethane molecules contribute to improved tensile strength, up to 250% compared to CWPU samples. Furthermore, the inclusion of lignin enhanced thermal stability, showcasing a 4.6% increase in thermal decomposition temperature compared to conventional samples and increased solvent resistance to ethanol. Moreover, CWPU-LX exhibits desirable characteristics such as protection against ultraviolet light and antibacterial properties. These unique properties can be attributed to the presence of the polyphenolic group and the three-dimensional structure of lignin, further highlighting the versatility and potential of this material in various application domains. The integration of lignin, a renewable and abundant resource, into CWPU-LX exemplifies the commitment to environmentally conscious practices and underscores the significance of greener materials in achieving a more sustainable future.

## 1. Introduction

Polyurethanes (PUs), which are synthesized through the step-growth polymerization of diisocyanate and polyol, are remarkably versatile and are utilized in a wide range of industrial applications. For example, PUs are used in foams [1,2], additives [3], elastomers [4], and coatings [5,6,7]. The exceptional and versatile mechanical, chemical, and physical properties of PUs are the main reasons for their widespread usage [8]. Although petroleum-based polyol is the material primarily used in commercial PU manufacturing, increasing awareness of environmental issues and concerns about petroleum scarcity have fueled research into alternative biomass resources [9]. Ongoing efforts are focused on investigating and developing sustainable PU production techniques using polyols derived from renewable biomass sources. For example, PU synthesis using renewable resources, such as vegetable-oil-derived polyols, has emerged as a promising approach. Fatty acids are commonly utilized as polyols. Various vegetable oils, including those derived from castor, soybean, cashew nut shell liquid, and cotton seed, have been investigated for their potential as sustainable polyol sources [10]. The use of vegetable oil as a raw material in the PU industry is gaining much attention because of the attractiveness of such oils as a reliable starting material that offers diverse opportunities for chemical transformations to develop novel products [11].

In many applications, PUs are used in conventional-solvent-based formulations that heavily rely on organic solvents. However, concerns about the generation of volatile organic compounds (VOCs) and hazardous air pollutants have led to an active investigation into developing environmentally friendly waterborne PU formulations that eliminate the need for organic solvents. Waterborne polyurethane (WPU) is a polymer that is dispersed in water and has minimal or no VOCs, making it advantageous because it enables stable storage and an eco-friendly solution [7]. WPU exhibits an exceptional adhesion ability to diverse substrates, including textile, leather, glass, and polymers. WPU also forms a transparent and odorless plastic coating that dries rapidly [12,13]. However, WPU has lower mechanical strength than solvent-based PUs, and hence, various additives such as fillers are used to enhance its properties.

Recently, lignin has gained attention as a filler to improve the physical properties and functionality of WPU [14]. Lignin is a renewable biomass obtained from plants, and has the second largest proportion after cellulose and it is being increasingly recognized for its abundance and cost-effectiveness. The structure of lignin is characterized by a three-dimensional network, in which three diverse phenylpropane units are interconnected through carbon–carbon bonds and ether linkages. It is a promising candidate for the formulation of new polymer composites because of its eco-friendly characteristics, dynamic functionality, and biodegradable properties [15]. For example, it has been incorporated into polybutylene adipate-co-terephthalate as a food packaging material because of its oxidant, antibacterial, and barrier functionalities [16]. Functionalized lignin was also employed to fabricate PU foam with an enhanced modulus and specific compressive strength [17].

Ongoing studies are exploring the integration of lignin into PU matrices, with a focus on enhancing flame retardancy, UV shielding, and the incorporation of antibacterial functionalities for a wide array of applications [18,19,20]. The influence of different types of lignin on the PU network was examined during the synthesis of PU by using commonly employed soft components such as polyether, polyethylene glycol, and polyester [21]. A study focused on synthesizing a bio-based PU coating resin by modifying vinylic acid to yield an isocyanate group, which subsequently reacted with the hydroxyl group of lignin to form a urethane group [22]. Furthermore, because of the antioxidant and UV-blocking properties of lignin, a peptide-delivery PU carrier was fabricated [23].

In this study, a cost-effective and environmentally sustainable approach was introduced for creating bio-based nanocomposites by incorporating lignin into castor-oil-based cationic waterborne polyurethane (CWPU-LX) through a straightforward blending procedure. By incorporating lignin into the polyurethane matrix using this efficient and reduced-solvent method, the resulting CWPU-LX showcases enhanced properties, making it a promising material for various applications while promoting a more sustainable approach to waterborne polyurethane synthesis.

## 2. Materials and Methods

### 2.1. Materials

The bio-polyol used in this study was castor oil (CO), which was procured from Alfa Aesar (Ward Hill, MA, USA). Lignin in the form of sulfonic acid calcium salt was purchased from Sigma-Aldrich (water soluble, contains 5 wt% Ca, impurities <5 wt%, St. Louis, MO, USA). The molecular weights of CO and lignin were determined by gel permeation chromatography (GPC) analysis as 1625 and 2029 g mol^−1^, respectively. Polytetramethylene ether glycol 2000 (PTMEG) was purchased from Korea PTG Co., Ltd (Ulsan, Republic of Korea). Isophorone diisocyanate (IPDI), N-methyldiethanolamine (MDEA), and diethanolamine (DEA) were procured from Samchun Chemical (Seoul, Republic of Korea). Dibutyltin didodecanoate (DBTDL) was purchased from Junsei Chemical (Tokyo, Japan). Methyl ethyl ketone (MEK) and acetic acid (AA) were obtained from Daejung Chemical (Busan, Republic of Korea). All materials were used as received without additional purification.

### 2.2. Synthesis of CWPU-LX

A dry three-neck flask was used to homogenize IPDI and MDEA using a mechanical stirrer at 50 °C for 50 min. Subsequently, CO was added to the mixture, and the reaction proceeded at 65 °C for 30 min. A single drop of DBTDL was added to treat the mixture. DEA was incorporated to extend the prepolymer chain, and MEK was added to lower the viscosity of the mixture. The resulting mixture was stirred for 3 h at 65 °C. Upon cooling to 30 °C, AA was introduced as a neutralizer, and the mixture was stirred for 30 min. The mixture was then dispersed with aqueous solutions at a stirring speed of 400 rpm for a period of 12 h. Finally, an environmentally friendly castor-oil-based cationic waterborne PU (CWPU) was obtained. In the preparation of the aqueous solutions, various concentrations of lignin (0, 1, 3, 5, and 7 wt% to the solid contents of CWPU) were measured and dispersed in 100 mL of deionized water. The interaction between the dispersed urethane molecules and lignin molecules within the aqueous solution led to the formation of stable emulsions. Subsequently, a rotary evaporator was used to remove any remaining MEK present in the reactor; thus, CWPU with a total solid content of 8–10 wt% was obtained. Based on the amount of lignin added, the resulting CWPU samples were denoted as CWPU-LX, where X represented the percentage of lignin in the CWPU. The CWPU-LX films were obtained by the solvent-casting method in a polytetrafluoroethylene mold. For comparison, PTMEG-L0 was synthesized using PTMEG as the polyol following the same procedure as described above. The molar ratios of the chemicals are listed in Table 1.

### 2.3. Characterization

The molecular weight and distribution of the prepared bio-based CWPU samples were analyzed by the GPC using the Alliance e2695 instrument manufactured by Waters Corp., Milford, MA, USA. The flow rate was set to 1 mL min^−1^ and the temperature was maintained at 35 °C. Tetrahydrofuran (THF) was used as the mobile-phase solvent to determine the molecular weight of CWPU-LX. The surface morphology of CWPU-LX was examined by field-emission scanning electron microscopy and energy-dispersive spectroscopy (SEM-EDS, SU8230, Hitachi, Tokyo, Japan) with a working distance of 15 mm and an acceleration voltage of 15 kV. Fourier transform infrared spectroscopy (FT-IR; Nicolet 380, Thermo Fisher Scientific, Waltham, MA, USA) was performed using the attenuated total reflection method (Smart iTR ZnSe) to determine the chemical structure of lignin and CWPU. The thermal stability of the samples was analyzed using a Q500 TGA thermal analyzer (TA, Robbinsville, NJ, USA) in a nitrogen atmosphere with a heating rate of 10 °C min^−1^ and a temperature range of 25–800 °C. Dynamic mechanical analysis was conducted to investigate the thermomechanical properties of the CWPU films. The film samples were subjected to cyclic loading with a frequency of 1 Hz over a temperature range of −50–70 °C with a temperature variation rate of 5 °C min^−1^. The dimensions of the specimens were 40 × 10 × 0.4 mm (length × width × thickness). The mechanical properties of the prepared CWPU films were evaluated using a universal testing machine (UTM, OTT003, Oriental TM, Ansan, Republic of Korea) with a 20 kgf load cell and an extension rate of 10 mm min^−1^. A minimum of five specimens were tested for each sample. X-ray diffraction (XRD) experiments were performed using a Panalytical Empyrean X-ray diffractometer with CuKα radiation (λ = 0.15418 nm). The water contact angle was measured at room temperature (25 °C) using the static sessile drop method with a contact angle measurement system (Holmarc, Kerala, India). For drop shape analysis, the drop analysis plugin available in ImageJ software was used. The optical absorbance of the CWPU-LX films was measured for the wavelength range of 190–800 nm using an OPTIZEN POP UV/visible light (VIS) spectrophotometer (Lklab, Namyangju-si, Republic of Korea).

### 2.4. Determining the Hydroxyl Content of Castor Oil

The hydroxyl content of CO was determined in accordance with ASTM D 1957. The hydroxyl value was calculated using the following equation:

(1) $$\mathrm{Hydroxyl}\;\mathrm{value}=\frac{B+\left(SA/C\right)-V}{S}\times N\times 56.1$$

where *A* represents the volume (in mL) of KOH solution required for the titration of the acid value; *B* represents the volume (in mL) of KOH solution required for the titration of the reagent blank; *C* represents the mass (in g) of the sample used for the acid value; *V* represents the volume (in mL) of KOH solution required for the titration of the acetylated specimen; and *S* represents the mass (in g) of the sample used for acetylation. The hydroxyl value of CO was determined to be 178.9 mg KOH g^−1^ and 3.19 mmol g^−1^.

### 2.5. Antibacterial Properties Test

The antibacterial properties of the resulting CWPU-LX films against Gram-positive bacteria (*Staphylococcus aureus*) and Gram-negative bacteria (*Escherichia coli*) were evaluated using a modified swab inoculation assay [24]. The objective of this investigation was to rigorously assess the bacterial attachment and survival on the surface of CWPU-LX films. The bacteria were cultured in nutrient broth at 25 °C for 24 h, and a bacterial stock was prepared by diluting the cells to a concentration of 1 × 10^4^ colony-forming units per milliliter (CFU mL^−1^) using nutrient broth. A 300 μL volume of the bacterial stock was inoculated onto the control and CWPU-LX films, and spread on the film surfaces using a cotton swab. The films were incubated at 25 °C for 24 h under a relative humidity > 90%. The samples were carefully transferred onto the agar plates, ensuring proper contact, and then incubated at 25 °C for 4 days. The antibacterial efficacy was determined by counting the CFUs of viable bacteria on the agar plates using Image J.

## 3. Results and Discussion

**Scheme 1:**
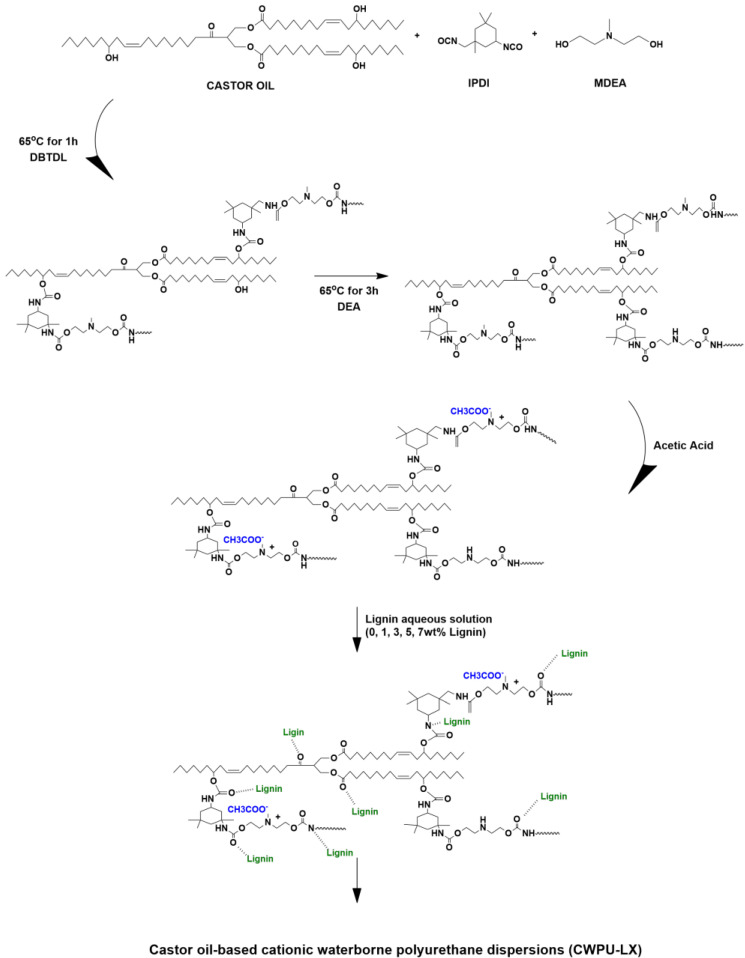
Synthesis mechanism of a castor oil (CO)-based cationic waterborne polyurethane with lignin (CWPU-LX).

The synthetic mechanism of CWPU-LX is illustrated in Scheme 1. The network structure of CWPU is derived from the reaction of the hydroxyl groups of CO and MDEA with the isocyanate group of IPDI, resulting in the formation of isocyanate-terminated urethane prepolymers. These prepolymers react with DEA (which is used as a chain extender) and form PU. PU is neutralized with AA and an aqueous lignin solution is added to it to obtain CWPU-LX. The incorporation of lignin between PU molecules leads to the formation of stable CWPU-LX emulsions. The presence of lignin facilitates diverse interactions and enhances the mechanical properties of the material via hydrogen bonding and dynamic interactions between PU molecules and lignin. The molecular weights of the synthesized CWPU-LX (as characterized by GPC) are summarized in Table 2. The polydispersity index (PDI) values of all synthesized CWPU lie between 2 and 3.5, while the number average molecular weight (Mn) values range from 7000 to 15,000 and the weight average molecular weight (Mw) values range from 20,000 to 35,000.

The experimental approach in this study significantly deviates from the conventional waterborne polyurethane dispersion synthesis method. The typical method of polyurethane synthesis involves the initial reaction between polyol and isocyanate, resulting in the formation of a prepolymer, which is then further extended by the addition of an extender through a subsequent reaction. The use of CO, which contains three hydroxyl groups, in this particular study presents a distinct challenge compared to conventional polyols. The chain extension process with CO leads to a rapid increase in viscosity. Consequently, the addition of multiple solvents becomes necessary to reduce the viscosity. In this study, a different approach was adopted, where a short urethane chain was initially formed by combining MDEA and IPDI. Subsequently, a prepolymer was synthesized by reacting the short urethane chain with CO. The polyurethane was prepared through an additional chain extension reaction with DEA. The implementation of this method effectively reduces the solvent quantity required for CWPU-LX synthesis to approximately 50% of that used in traditional experimental procedures. This reduction in solvent consumption contributes to the production of an eco-friendly polyurethane, emphasizing the sustainability of the synthesis process.

**Figure 1:**
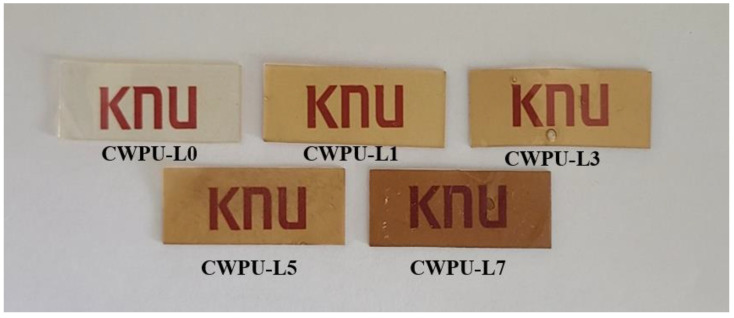
Photographs of the CWPU-LX films.

Figure 1 shows a photograph of the prepared CWPU-LX film, which is translucent. With increasing lignin content, the color of the film changes from light yellow to brown. Notably, the integration of lignin into the CWPU film does not produce any observable particulate entities. The SEM-EDS analysis (Figure S1, Supplementary Material) shows that the absence of observable particulate entities or significant agglomeration in specific regions of the film. The absence of observable particulate entities or significant agglomeration in specific regions of the film, as indicated by the results shown in the images, supported our assertion of good lignin dispersion.

**Figure 2:**
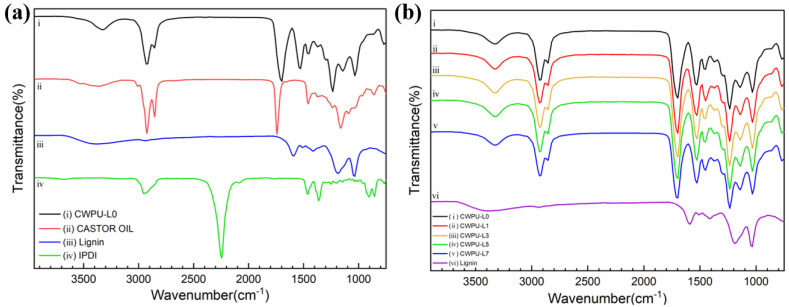
Fourier transform infrared (FT-IR) spectra of (**a**) raw materials and (**b**) CWPU-LX.

The FT-IR spectra of the materials and the prepared CWPU-LX are shown in Figure 2. IPDI reveals the appearance of a stretching vibration absorption peak attributed to the isocyanate groups at wavenumbers in the range of 2280–2300 cm^−1^. The spectrum of CO exhibits absorption peaks at 2930 and 2850 cm^−1^, which are assigned to the CH– and CH2 vibrations, respectively. In both the CO and lignin spectra, a broad stretching vibration peak associated with –OH is observed in the wavenumber range of 3300–3500 cm^−1^. The spectrum of lignin shows absorption peaks at 1412, 1512, and 1600 cm^−1^, which correspond to the stretching vibrations of aromatic-ring structures [25]. The C–O stretching vibration of the G (guaiacol) ring is assigned to the peak observed near 1190 cm^−1^, while the peak at around 1050 cm^−1^ is attributed to the S=O stretching of sulfonate groups in lignin [26]. Upon the formation of the urethane bond between polyol and isocyanate during the reaction, the isocyanate group corresponding to the 2280 cm^−1^ peak is no longer present in CWPU-LX. The FT-IR spectrum of CWPU-LX does not indicate any stretching vibration peaks corresponding to the –OH groups in the wavenumber range of 3300–3500 cm^−1^. Instead, a sharp peak associated with the stretching vibration of –NH in the carbamate groups appears at 3330 cm^−1^, confirming the successful formation of PU through the reaction between the polyol and isocyanate. Furthermore, the –C=O stretching vibration and –C–O–C– bond of the urethane linkages in CWPU are assigned to the peaks observed at 1697 and 1234 cm^−1^, respectively [27]. The effect of the lignin ratio on peak intensity is negligible, likely because of the structural characteristics of lignin (such as steric hindrance), which restrict the influence of its hydroxyl group and hinder the observation of peak intensity changes with varying lignin amounts [28]. Moreover, the overlapping of peaks can complicate the accurate observation of intensity changes, making it challenging to pinpoint specific alterations directly attributable to lignin content. This also suggests that lignin does not form a direct chemical bond among the PU molecules.

**Figure 3:**
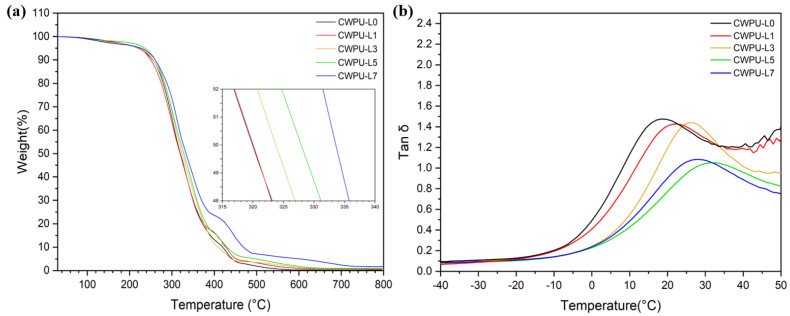
Thermal properties of CWPU-LX: (**a**) thermogravimetric analysis (TGA) curves and (**b**) DMA result (tan δ).

The thermal properties of the prepared CWPU-LX are shown in Figure 3 and Table 3. The figure shows the parameters T_onset_, T_50_, T_max_, and T_g_. The thermal stability of CWPU-LX is influenced by the characteristics of the soft and hard segments and their intermolecular interactions [29]. The decomposition of CWPU-LX occurs primarily through the breakdown of the urethane groups at temperatures of 200–300 °C, while CO and lignin undergo decomposition at higher temperatures, specifically between 300 °C and 400 °C [30,31]. The T_50_ value increases as the lignin content increases, indicating that the presence of lignin positively affects the rate of weight loss during decomposition. The observed increase in T50 can be attributed to the enhanced crosslinking density of CWPU-LX and the filler effect of lignin [25,32]. Figure 3b shows that the correlation between lignin content and T_g_ of CWPU-LX can be evaluated from the loss factor analysis in DMA. An increase in lignin content leads to a corresponding increase in the T_g_ value of CWPU-LX. With higher lignin concentrations, the tan δ peak of CWPU-LX becomes broader, and its maximum value decreases, indicating an increase in the crosslinking densities. This suggests that the higher lignin content leads to an increase in crosslinking density, resulting in decreased energy dispersion along the polymer chain [25,33].

**Figure 4:**
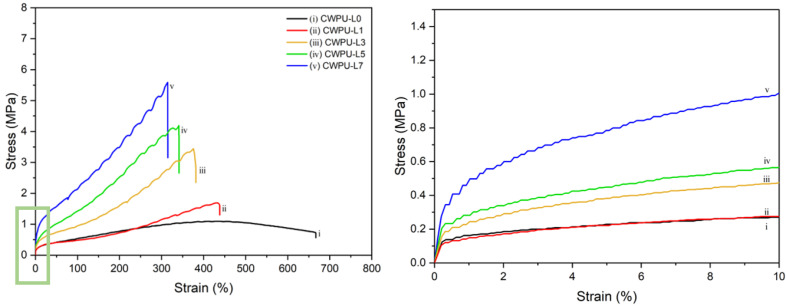
Stress–strain curves and summary of analysis of the CWPU-LX films (The figure on the right is an enlarged view of the green square area in the left S-S curve).

The mechanical properties of the CWPU-LX films are shown in Figure 4 and Table 4. With an increase in the lignin concentration, the CWPU-LX films exhibit improved mechanical performance. The lignin content of CWPU-LX and its tensile strength are positively correlated. The tensile strength increases from 1.10 MPa at 0 wt% to 5.57 MPa at 7 wt%, while the elongation at break decreases from 666.9% to 314.3%. The improved mechanical properties of the CWPU-LX films can be attributed to the increase in the strength of the interactions between PU and lignin molecules [25]. Lignin fills the gaps in CWPU-LX, thus increasing the free volume of the polymer matrix and improving the density of the crosslinking. This results in improved mechanical properties. The introduction of lignin into the polymer matrix alters the arrangement of the polymer chains, resulting in stronger hydrogen bonds than in the initial state of the polymer [34]. As the amount of lignin increases, the strength of these interactions also increases. However, at higher concentrations, factors such as steric hindrance can cause weaker interactions [35,36].

**Figure 5:**
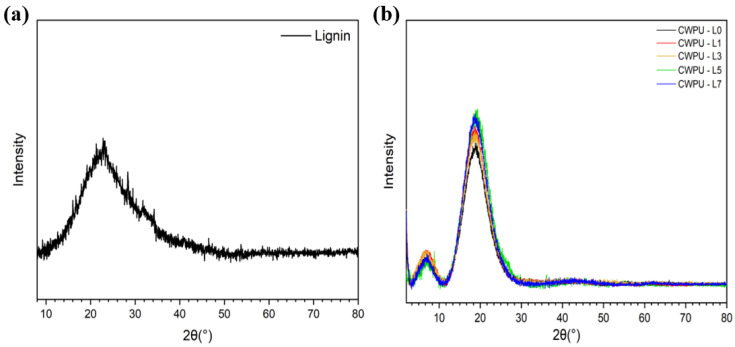
X-ray diffraction (XRD) analysis results of (**a**) lignin and (**b**) CWPU-LX.

The influence of lignin on the crystallization behavior of PU elastomers was examined by XRD analysis. Figure 5a shows the broad scattering peak in the diffraction curves of lignin at approximately 2*θ* = 21°–23° and Figure 5b shows the broad scattering peaks of CWPU-LX at approximately 2*θ* = 8° and 18°–19°. The peak at 2*θ* = 8° indicates a crystalline region. The peak at 2θ = 18°–19° suggests that CWPU-LX has an amorphous structure, and the increasing sharpness of this peak indicates the development of ordered amorphous regions [37,38]. The concentration of lignin affects the formation of the ordered amorphous regions in the PU molecular chain, as evidenced by the sharper scattering peak at 2*θ* = 18°–19° with increasing lignin content. The degree of crystallinity can be estimated through the full width at half maximum (FWHM) values of the XRD peak. As shown in Table 5, higher lignin ratios result in higher FWHM values around 2*θ* = 19°; this result indicates a random shape in the ordered amorphous region because of hydrogen bonding between the lignin and PU molecules. Addition of lignin to the PU matrix results in additional hydrogen bonds, which enhances the constraint and orientation of the chain segments during stretching; therefore, the material is drastically self-reinforced through strain-induced crystallization [39]. Furthermore, in agreement with the S–S curve results shown in Figure 4, an increase in the content of lignin induces a transition from an amorphous region to an ordered amorphous region, leading to enhanced tensile strength and reduced elongation at break. These effects are attributed to the structural characteristics of the polyphenol in lignin and to chemical effects such as hydrogen bonding. Thus, incorporating lignin in PU elastomers leads to the formation of dynamic molecular chains and ultimately improves the mechanical properties of the material [40].

**Figure 6:**
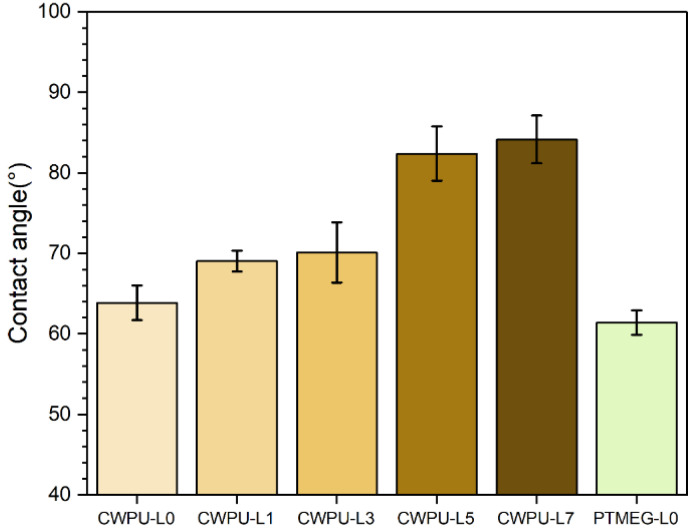
Water contact angle of PTMEG-L0 and CWPU-LX.

The results of the water contact angle test reveal that the hydrophilicity of the films is influenced by lignin concentration; higher lignin ratios result in higher contact angles. The enhancement of the hydrogen bond between the lignin and CWPU, as the amount of lignin increases, contributes to the compactness of CWPU-LX, limiting water penetration and reducing surface wetting [25,41]. Figure 6 shows that PTMEG-L0 has a higher hydrophilicity than CWPU-L0. Generally, the crosslink density between CO and isocyanate is higher than that of the petroleum-based polyol because of the disparity in the number of hydroxyl groups present in their respective molecules. Petroleum-based polyol contains two hydroxyl groups per molecule, whereas CO contains three hydroxyl groups; thus, CO has a higher crosslink density. The higher hydrophilicity of petroleum-based waterborne PU, compared to CWPU-L0, can be attributed to this difference in crosslink density [42,43].

**Figure 7:**
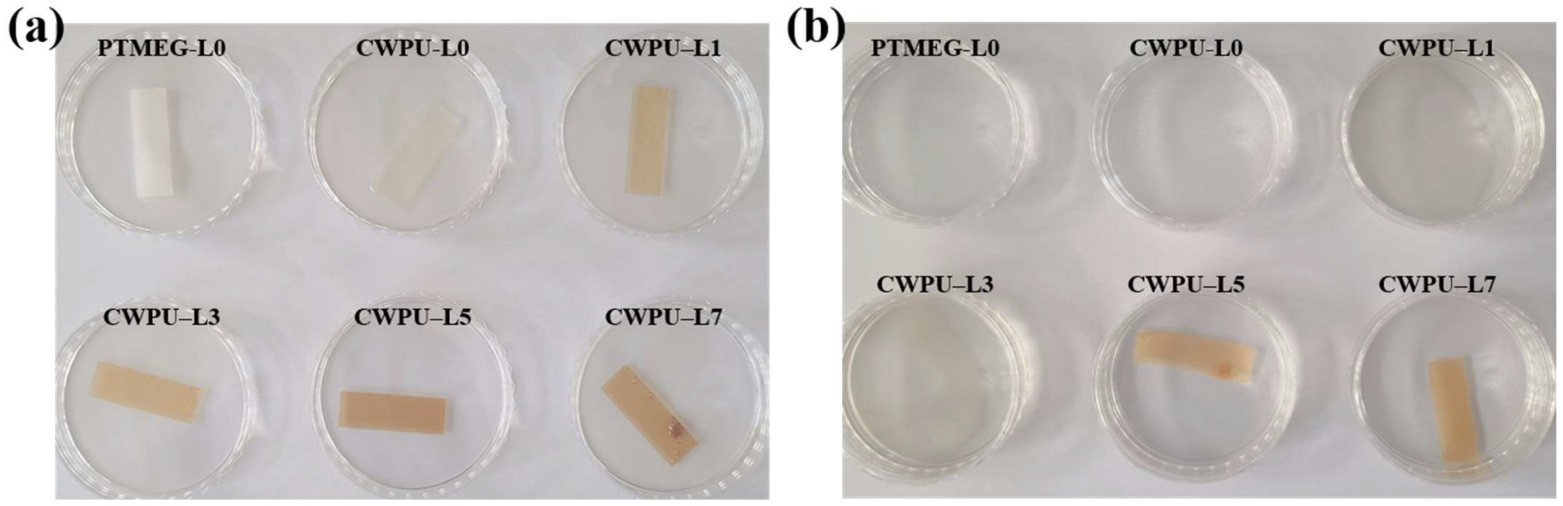
Ethanol resistance of PTMEG-L0 and CWPU-LX films (**a**) at 0 h and (**b**) after 10 h.

The ethanol resistance of CWPU-LX is shown in Figure 7. The ethanol resistance test revealed complete dissolution of CWPU-L0 and PTMEG PU after a 10 h exposure to ethanol. PTMEG-L0, CWPU-L0, CWPU-L1, and CWPU-L3 completely dissolve in ethanol, leaving behind a minimal residual film. In contrast, CWPU-L5 and CWPU-L7 remain in the film form when exposed to ethanol; CWPU-L5 exhibits relatively more swelling than CWPU-L7. To synthesize PTMEG PU and CWPU-LX, we used MDEA and DEA with cationic ions as the extenders. Under the influence of cations, PTMEG-L0 and CWPU-L0 dissolve well in ethanol. As the MDEA content increases, the ethanol uptake increases accordingly because of the increased presence of hydrophilic ionic groups and crosslinking densities [29,30]. However, in the case of CWPU-LX, lignin enters into the voids of the urethane chain and interacts with the urethane chain. When the concentration of lignin increases, the interaction between lignin and the urethane chain becomes stronger, surpassing the strength of interaction between ethanol and the urethane chain. This enhanced interaction helps preserve the shape of the CWPU-LX film.

**Figure 8:**
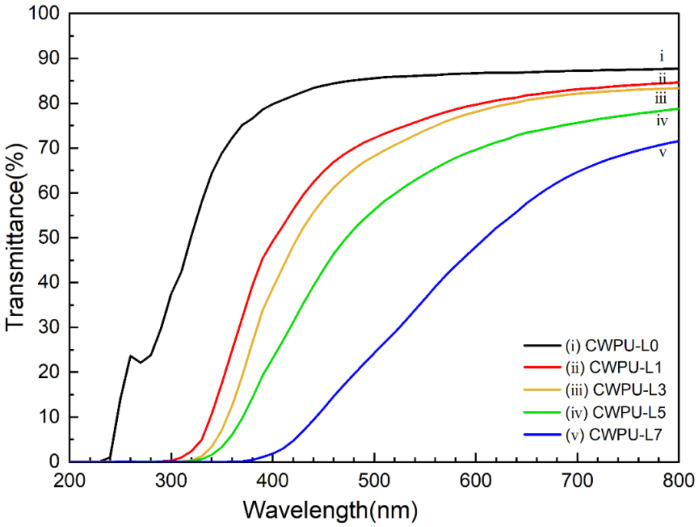
Ultraviolet (UV) transmittance spectra of CWPU-LX films with different lignin contents.

Because of the structure of lignin, it can effectively block UV radiation. Lignin contains UV-absorbing chromophore groups such as phenolics, ketones, quinoid structures, and intramolecular hydrogen bonds. Hence, lignin is highly desirable for several applications owing to its UV-blocking properties [44,45,46]. To achieve effective UV absorption, a higher concentration of lignin should be incorporated into CWPU-LX, as shown in Figure 8. The UV spectrum encompasses three regions: UV A, B, and C, with corresponding wavelength ranges of 320–400, 280–320, and 100–280 nm, respectively. The addition of lignin to CWPU-LX leads to enhanced UV-blocking capability. The CWPU-L1 film exhibits UV absorption up to 300 nm (UV-C). CWPU-L3, CWPU-L5, and CWPU-L7 can absorb UV wavelengths up to 315 nm (UV-B and C), 320 nm (UV-B and C), and 380 nm (UV-A, B, and C), respectively. As the lignin content increases relative to the PU solid content during the film fabrication process, the amount of lignin incorporated within the manufactured film increases as well. This higher lignin content significantly contributes to the elevated UV-blocking capabilities of the resulting film. Moreover, as depicted in Figure 1, the deepening color and increasing opacity of the film with higher lignin content are visually evident. This observation aligns with the phenomenon where heightened lignin content leads to a darker coloration and a gradual increase in opacity. Consequently, the overall transmittance in both the UV and visible regions decreases due to the higher absorption and scattering properties attributed to lignin-rich films.

**Figure 9:**
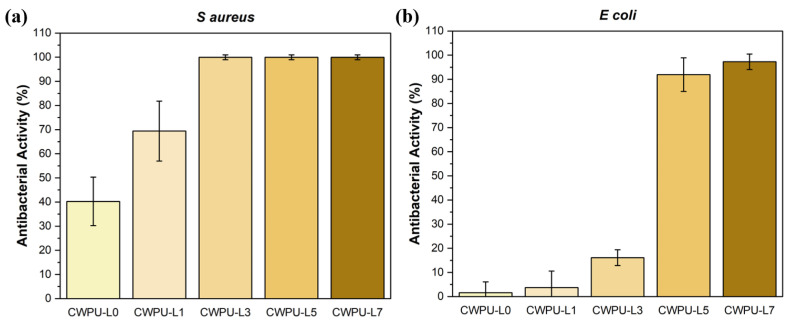
Antibacterial activity of CWPU-LX films with different lignin contents against (**a**) Gram-positive (*Staphylococcus aureus*) and (**b**) Gram-negative (*Escherichia coli*) bacteria.

The antibacterial activities of CWPU-LX against Gram-positive (*Staphylococcus aureus*) and Gram-negative (*Escherichia coli*) bacteria were analyzed by a modified swab inoculation assay. As shown in Figure 9, the antibacterial activity is positively correlated with the lignin content in the films. The introduction of lignin leads to a significant increase in antibacterial efficacy against *S. aureus*. Specifically, films CWPU-L3, CWPU-L5, and CWPU-L7 exhibit impressive antibacterial activities of 99.99%, while the activity of CWPU-L0 is 40.25%. Similarly, the antibacterial activity against *E. coli* show a substantial improvement, with CWPU-L7 demonstrating an activity of 97.27%, compared to 1.58% for CWPU-L0. This enhancement in antibacterial properties can be attributed to the synergistic effect of lignin and ammonium cations (MDEA) in the CWPU-LX samples. The unique three-dimensional structure and polyphenolic composition of lignin, containing phenolic hydroxyl and methoxy groups, have been recognized as essential factors contributing to its antibacterial activity [47,48,49]. Additionally, the use of ammonium cations, such as MDEA, as an extender in the WBPU synthesis contributed to the antibacterial effect. Their strong affinity towards bacterial cells, due to their positive charge, further contributed to the observed antibacterial effect [50,51]. The combination of lignin’s polyphenolic composition and the presence of ammonium cations contributed to the observed antibacterial effect. These findings highlight the potential of CWPU-LX films as sustainable and antimicrobial materials for various applications, including packaging and biomedical devices.

## 4. Conclusions

In this research, we present a novel and environmentally conscious method for the synthesis of lignin-incorporated castor-oil-based cationic waterborne polyurethane (CWPU-LX), which diverges significantly from conventional approaches. The modified synthesis route resulted in a significant reduction in solvent quantity required for CWPU-LX synthesis, lowering it to approximately 50% of that used in traditional experimental procedures. The reduction in solvent consumption highlights the eco-friendliness and sustainability of our synthesis process, aligning with the growing emphasis on green chemistry and environmentally friendly materials. By incorporating lignin into the polyurethane matrix using this efficient and reduced-solvent method, the resulting CWPU-LX showcases enhanced properties, making it a promising material for various applications while promoting a more sustainable approach to waterborne polyurethane synthesis. The dynamic interaction between lignin and PU molecules resulted in substantial enhancements in mechanical properties, including a notable elevated tensile strength from 1.10 MPa to 5.57 MPa. Additionally, this interaction manifested enhanced thermal stability, with an increase in thermal decomposition temperature from 319.19 °C to 333.78 °C. Furthermore, CWPU-LX exhibits exceptional attributes, such as UV-light protection and antibacterial properties, which can be attributed to the presence of the polyphenolic group and the unique three-dimensional structure of lignin. These findings underscore the versatility and potential of CWPU-LX for various applications like food packaging, coatings, and biomedical applications.

Our research not only introduces an innovative synthesis approach for advanced polyurethane materials but also promotes a more sustainable and eco-friendly direction in waterborne polyurethane production. The successful integration of lignin, a renewable and abundant resource, into CWPU-LX exemplifies our commitment to environmentally conscious practices, emphasizing the significance of greener materials in striving towards a more sustainable future.

## Figures and Tables

**Table 1:** Formulation of the bio-based polyurethane sample.

Sample	Polyol (func. mol)		IPDI (func. mol)	Chain Extender (func. mol)		Lignin (wt%)	Content of Bio-Based Component (wt%)
	CO	PTMEG		MDEA	DEA		
CWPU-L0	1	-	2.5	0.6	0.1	0	47.35%
CWPU-L1	1	-				1	47.89%
CWPU-L3	1	-				3	48.92%
CWPU-L5	1	-				5	49.85%
CWPU-L7	1	-				7	50.81%
PTMEG-L0	-	1				0	0.00%

**Table 2:** Gel permeation chromatography (GPC) results of the prepared polytetramethylene ether glycol (PTMEG-L0) and CWPU-LX.

Sample	Mw	Mn	PDI
CWPU-L0	21,781	7320	2.97
CWPU-L1	22,980	7941	2.89
CWPU-L3	32,604	14,716	2.22
CWPU-L5	24,937	7107	3.5
CWPU-L7	35,102	10,181	3.44
PTMEG-L0	55,831	29,721	1.87

**Table 3:** Thermal properties of CWPU-LX: summary of the thermal analysis results.

Sample	Thermal Properties			
	TGA Analysis (°C)			DMA Analysis (°C)
	T_onset_	T_50_	T_max_	T_g_
CWPU-L0	263.94	319.19	303.59	18.41
CWPU-L1	256.37	319.92	300.69	22.44
CWPU-L3	256.27	323.77	305.86	25.76
CWPU-L5	260.07	327.95	304.11	31.67
CWPU-L7	261.04	333.78	297.94	27.44

**Table 4:** Summary of mechanical properties of the CWPU-LX films.

Sample	Tensile Strength (MPa)	Elongation at Break (%)
CWPU-L0	1.10	666.9
CWPU-L1	1.69	438.2
CWPU-L3	3.43	375.2
CWPU-L5	4.18	340.9
CWPU-L7	5.57	314.3

**Table 5:** FWHM analysis of CWPU-LX.

Samples	FWHM ^a^
CWPU-L0	6.5504 ± 0.03
CWPU-L1	6.5573 ± 0.03
CWPU-L3	6.6449 ± 0.03
CWPU-L5	6.6445 ± 0.05
CWPU-L7	6.7008 ± 0.02

^a^ Full width at half maximum.

## Data Availability

The data presented in this study are available upon request from the corresponding author.

## Supplementary Materials

The following supporting information can be downloaded at: https://www.mdpi.com/article/10.3390/polym15193987/s1, Figure S1: Scanning electron microscopy and energy dispersive spectroscopy (SEM-EDS) images of the CWPU-LX films.
