# Supplementary material for: Sustainable Strategies for Synthesizing Lignin-Incorporated Bio-Based Waterborne Polyurethane with Tunable Characteristics
Source: Polymers (Basel). 2023 Oct 4;15(19):3987. doi: 10.3390/polym15193987 (PMC10575038; doi:10.3390/polym15193987)
Supplement: Supplementary file 1 [file polymers-15-03987-s001.zip › polymers-2625520-supplementary.pdf]

polymers

MDPI

Supplementary Materials: Sustainable Strategies for Synthesizing Lignin-Incorporated Bio-Based Waterborne Polyurethane with Tunable Characteristics

Bo Min Kim, Jin Sil Choi, Sunjin Jang, Hyeji Park, Seung Yeol Lee, Joonhoo Jung and Jaehyeung Park

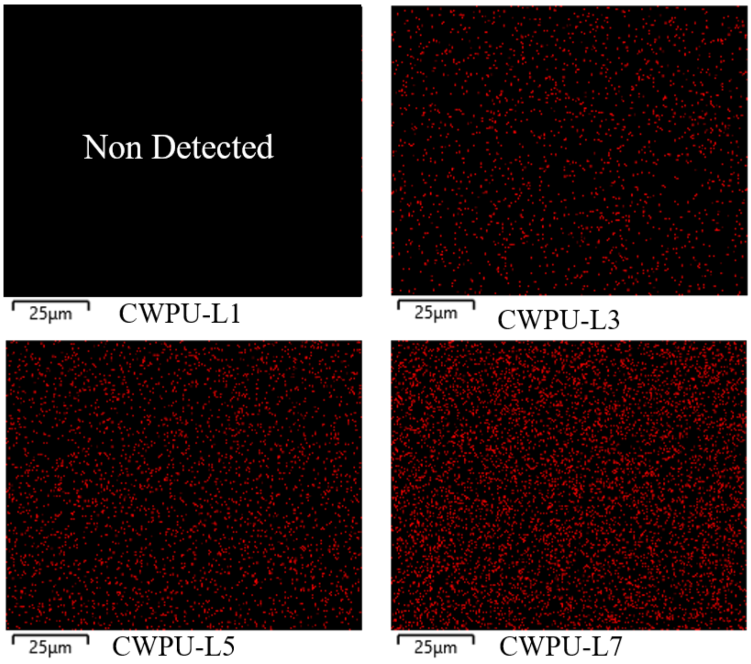

Figure S1. Scanning electron microscopy and energy dispersive spectroscopy (SEM-EDS) images of the CWPU-LX films.

The red-marked portion highlights the presence of sulfur within the lignin structure. However, the EDS analysis could not detect the presence of lignin in the CWPU-L1 sample because the lignin content was too low and exceeded the resolution limitations of the technique.
